# An Evaluation of the Cytotoxicity and Safety Profile of Usnic Acid for a Broad Panel of Human Cancers and Normal Cells with Respect to Its Enantiospecificity

**DOI:** 10.3390/molecules30142964

**Published:** 2025-07-14

**Authors:** Gabriela Siedlarczyk, Paweł Paśko, Agnieszka Galanty

**Affiliations:** 1 Department of Pharmacognosy, Jagiellonian University Medical College, Medyczna 9, 30-688 Kraków, Poland; gabriela.siedlarczyk@alumni.uj.edu.pl; 2 Department of Food Chemistry and Nutrition, Jagiellonian University Medical College, Medyczna 9, 30-688 Kraków, Poland; p.pasko@uj.edu.pl

**Keywords:** UA, cytotoxic, enantiomers, selectivity, toxicity

## Abstract

Chirality plays a key role in the effectiveness and toxicity of bioactive compounds. Usnic acid (UA), a lichen metabolite, exists as two enantiomers. Despite numerous studies on its biological properties, enantioselective aspects remain poorly recognized. This study assessed the cytotoxicity of UA enantiomers against colon, prostate, thyroid, brain, and breast cancer cell lines, as well as non-cancerous cells. Cell viability was determined by the MTT assay after 24, 48, and 72 h. Colon cancer HCT116 cells were the most sensitive (IC_50_ ~10 µg/mL, 72 h), with no enantiomeric dominance. In prostate cancer PC3 cells, (+)-UA was more effective. Moderate cytotoxic effect was noted for thyroid cancer cells; however, this was evaluated for the first time. MDA-MB-231 breast cancer cells were strongly affected (IC_50_ 15.8 and 20.2 µg/mL for (+)- and (−)-UA, 72 h), as compared to MCF7 cells. Brain cancer cells were the least affected, as so were normal astrocytes. UA had no effect on normal colon epithelial cells but showed moderate toxicity in prostate, thyroid, and breast cells. To conclude, the overall cytotoxicity of (+)-UA was stronger than its (−)-enantiomer, while the latter compound was more toxic to normal cells. These findings highlight the advantage of (+)-UA, especially in chemopreventive strategies.

## 1. Introduction

**Figure 1:**
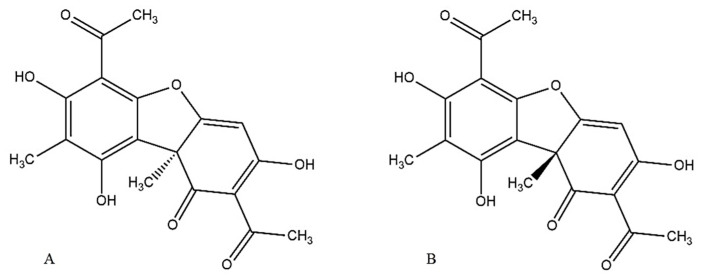
Structures of (−)-usnic acid (**A**) and (+)-usnic acid (**B**).

Chirality plays a crucial role in the effectiveness and toxicity of bioactive compounds. Usnic acid (UA), a dibenzofuran derivative, is synthesized by various lichen species in the form of two enantiomers that differ in the spatial orientation of a methyl group at the chiral center (Figure 1). The compound is found in high amounts (up to 10%) in several lichen species (e.g., *Cladonia*, *Flavocetraria*) and can be isolated relatively easily from the lichen matrix, what, together with its broad spectrum of biological activities, has been attracting significant scientific interest for years. Among the reported biological and pharmacological activities, cytotoxic, anti-inflammatory, and antimicrobial seem to be most promising, and all these aspects have been summarized in detail by a number of recent review articles [1,2,3,4]. Despite the large number of publications reporting the biological potential of UA, the enantioselective aspects of its activity and toxicity remain poorly characterized, as most research has focused on the (+)-enantiomer, leaving the biological profile of the (−)-form relatively underexplored [1].

The biological activity and toxicity of chiral compounds can vary significantly between the enantiomers. Enantioselectivity has been proven for a number of drugs used in the therapy of hypertension and bacterial infections, for example, or as anesthetics [5]. One of the most notable cases is thalidomide, where the (*S*)-isomer is associated with teratogenicity, in contrast to the (*R*)-isomer [6]. As for natural products, limited data on enantiospecificity exists, mainly including the differences in the effectiveness and toxicity of some monoterpenes enantiomers (e.g., pinene, limonene) or plant alkaloids (e.g., quinine) [7,8,9].

Comparative studies on the two enantiomers of UA are scarce. The prevalence of (+)-UA has been proven for its cytotoxic effects against some cancer cell lines [10,11,12]. In contrast, (−)-UA has demonstrated stronger antiviral [13,14], insecticidal [15], and phytotoxic activities [16]. In terms of toxicity, (−)-UA appears to exhibit greater genotoxicity [17], allergenic potential [18], and toxic effects on human skin keratinocytes [19] than its (+)-enantiomer. These findings suggest that the biological activity and toxicity of UA may be enantiomer-dependent and context-specific; however, due to some discrepancies, this issue needs further study.

The above-mentioned ambiguities concerning UA enantiomers inspired us to compare the cytotoxic potential of the enantiomers against a broad panel of human cancer cell lines, representing different tissues and stages of malignancy. Additionally, the study aimed to verify the toxicity of UA enantiomers on non-cancerous cells, which were included in the study.

## 2. Results and Discussion

The cytotoxic potential of UA enantiomers was evaluated across a diverse panel of human cancer cell lines, representing various tissue origins and differing in malignancy and metastatic potential. This approach was chosen to better reflect the phenotypic heterogeneity and complex nature of cancer. The cell lines were grouped into panels based on their tissue origin, including colon, prostate, thyroid, brain, and breast cancers. Additionally, data from our previously published study on the effects of UA enantiomers on melanoma cell lines [12] were included to provide a broader spectrum of results. To facilitate comparison with the findings from other studies, where IC_50_ values were reported in different concentration units, we recalculated those values (shown in parentheses) to ensure consistency and ease of comparison.

### 2.1. Cytotoxic Effect of UA Enantiomers on Colon Cancer Cells

**Figure 2:**
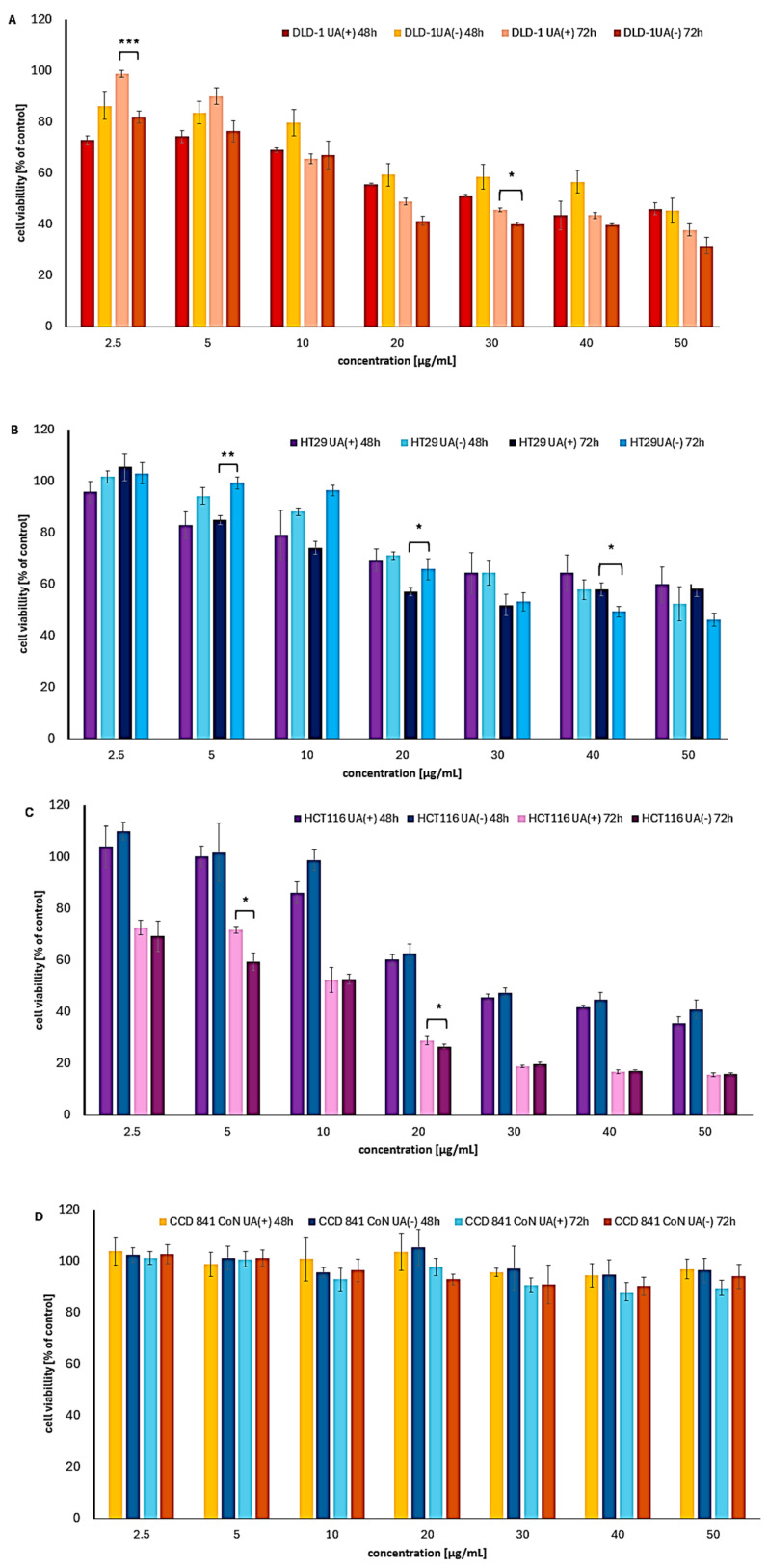
The cytotoxic impact of (+)- and (−)-UA on the viability of (**A**) DLD-1, (**B**) HT29, (**C**) HCT116 colon cancer cells, and (**D**) CCD 841 CoN normal colon cells after 48 and 72 h of exposure. Statistical analysis was performed by one-way analysis of variance (ANOVA), followed by Tukey’s post hoc test, with *p* ≤ 0.05 indicating statistically significant differences. Statistical differences between the enantiomers within the same concentration and exposure time are marked with black clamps, with *p* < 0.05 (*), *p* < 0.01 (**), and *p* < 0.001 (***).

After 24 h of treatment, the cytotoxicity of UA enantiomers was relatively weak, with cell viability rarely dropping below 70% (Table S1, Supplementary Materials). Both compounds exhibited variable cytotoxic effects on colon cancer cell lines, with the most pronounced decrease in cell viability observed after extended incubation periods of 48 and 72 h (Table 1, Figure 2). A clear dose-dependent response was noted, with increasing cytotoxicity observed at concentrations above 20 µg/mL for both enantiomers.

However, the response to UA treatment varied significantly among the different colon cancer cell lines. The most spectacular results were obtained for the HCT116 cell line, particularly after 72 h of exposure, where the IC_50_ values for both enantiomers were approximately 10 µg/mL. Notably, no significant difference in the potency was observed between the two enantiomers. This finding is especially important given the highly aggressive nature of the HCT116 cell line. Furthermore, the IC_50_ values for HCT116 after 72 h were markedly lower compared to those observed in the other colon cancer cell lines included in the study.

In contrast, a predominance of (+)-UA over (−)-UA was observed in DLD1 cancer cells after 48 h of treatment, with IC_50_ values of 26.1 and 44.3 µg/mL, respectively. Interestingly, this trend reversed after 72 h, when (−)-UA demonstrated greater cytotoxicity. Despite these fluctuations, both enantiomers exhibited strong cytotoxic effects against DLD1 cells, which are known for their high metastatic potential and represent an advanced stage of colon cancer. Among the tested cell lines, HT29 cells—derived from a primary tumor—were the most resistant to the compounds. An IC_50_ value was achieved only after 72 h of treatment with (−)-UA. In this case, (+)-UA showed slightly better efficacy than (−)-UA, but only at lower concentrations.

To date, no direct comparison of the cytotoxic activity of both UA enantiomers against colon cancer cells has been reported. Most existing studies focus exclusively on (+)-UA. The only exception is a study examining the effect of (−)-UA on HT29 cells [20], which reported an IC_50_ of 55 µM (equivalent to 18.9 µg/mL) after 48 h—contrasting with our findings. Other studies investigating (+)-UA in HT29 cells have reported moderate cytotoxicity, with IC_50_ values of 70 and 99.7 µM (24.1 and 34.3 µg/mL, respectively) [21,22]. For HCT116 and DLD1 cells, our results for (+)-UA are comparable to, or even more favorable than, those previously reported. For instance, Ref. [21] reports IC_50_ values of 100 µM (34.4 µg/mL) for both cell lines after 48 h, while [22] reports an IC_50_ of 143 µM (49.24 µg/mL) for HCT116 cells after 72 h. Notably, our study is the first to report the cytotoxic effects of (−)-UA on DLD1 and HCT116 cells.

### 2.2. Cytotoxic Effect of UA Enantiomers to Prostate Cancer Cells

**Figure 3:**
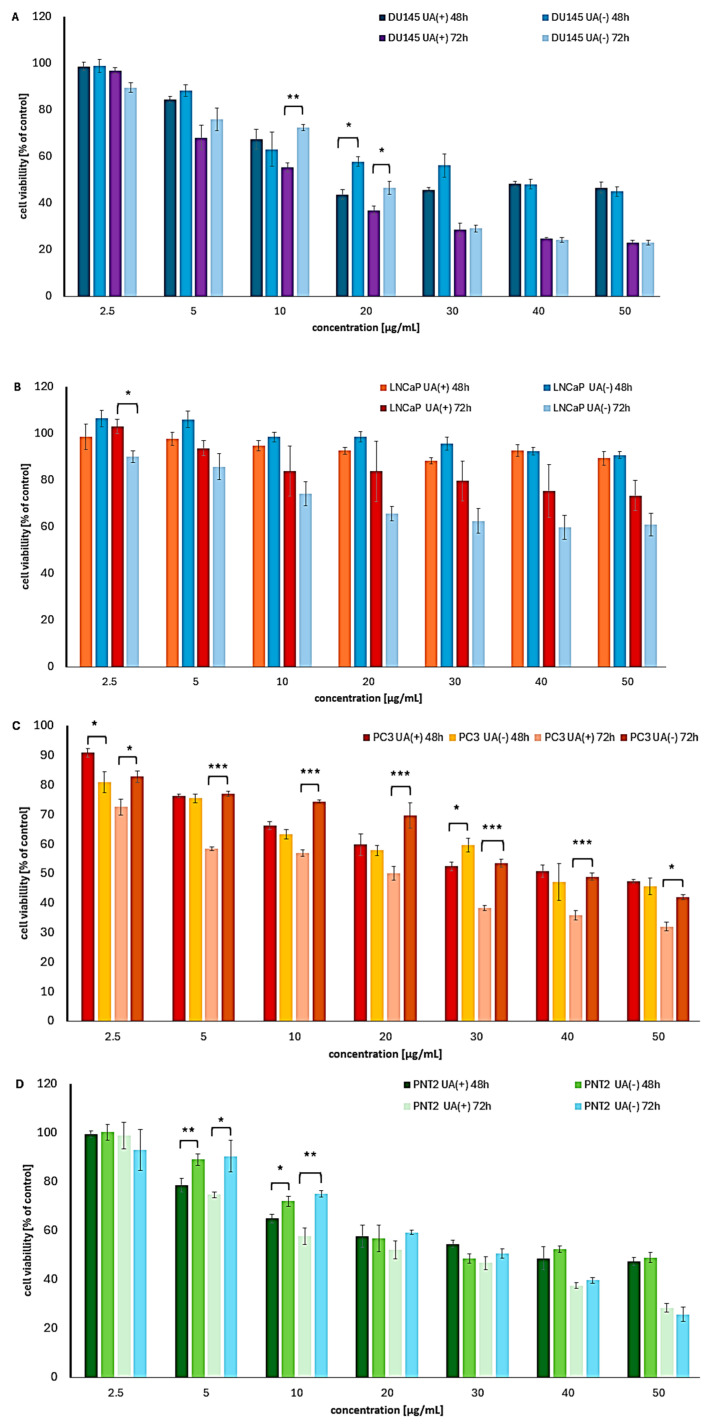
The cytotoxic impact of (+)- and (−)-UA on the viability of (**A**) DU145, (**B**) LNCaP, (**C**) PC3 prostate cancer cells, and (**D**) PNT2 normal prostate cells after 48 and 72 h of exposure. Statistical analysis was performed by one-way analysis of variance (ANOVA), followed by Tukey’s post hoc test, with *p* ≤ 0.05 indicating statistically significant differences. Statistical differences between the enantiomers within the same concentration and exposure time are marked with black clamps, with *p* < 0.05 (*), *p* < 0.01 (**), and *p* < 0.001 (***).

UA enantiomers exhibited variable cytotoxic effects on the tested prostate cancer cell lines, with the most notable impact on cell viability observed after prolonged incubation periods of 48 and 72 h (Table 2, Figure 3). After 24 h of treatment, both compounds showed only weak cytotoxicity, with cell viability remaining above 60% even at the highest tested concentration (Table S1).

Among the prostate cancer cell lines examined, the androgen-independent DU145 cells were the most sensitive to UA enantiomers. A stronger effect was observed for (+)-UA, particularly after 48 h of treatment and at concentrations below 30 µg/mL. Interestingly, a time-dependent cytotoxic response was noted only for (−)-UA.

Interestingly, at higher concentrations and after 72 h of incubation, no significant differences in the activity of the enantiomers were observed. Both compounds showed slightly weaker activity against the highly metastatic PC3 cells, compared to DU145 cells, with the most notable increase in cytotoxicity occurring after 72 h. Once again, (+)-UA demonstrated greater potency, with statistically significant differences across the entire tested concentration range after 72 h.

Among the prostate cancer cell lines tested, the androgen-dependent LNCaP cells were the most resistant. Notably, in this case, (−)-UA showed a clear advantage after 72 h of treatment, although the differences were not statistically significant.

To date, the cytotoxic potential of UA enantiomers has been directly compared in only two studies—on DU145 and PC3 cells—while no such comparison has been reported for the LNCaP cell line. In one study, (−)-UA exhibited higher cytotoxicity than (+)-UA in DU145 cells, with IC_50_ values of 57.4 µM vs. 45.9 µM (equivalent to 19.7 vs. 15.8 µg/mL) after 72 h [23], which contrasts with our findings. For PC3 cells, both enantiomers showed comparable activity, with IC_50_ values exceeding 10 µM (3.4 µg/mL), regardless of the exposure time [24].

On the other hand, our results for DU145 cells treated with (+)-UA after 48 h are consistent with a recently published study, which reported an IC_50_ of 42.15 µM (14.5 µg/mL) [25]. The only available report on the cytotoxic effect of UA enantiomers on LNCaP cells indicates an IC_50_ of 77.5 µM (26.7 µg/mL) for (+)-UA after 48 h of exposure [26], which is contrary to our observations.

### 2.3. Cytotoxic Effect of UA Enantiomers on Thyroid Cancer Cells

**Figure 4:**
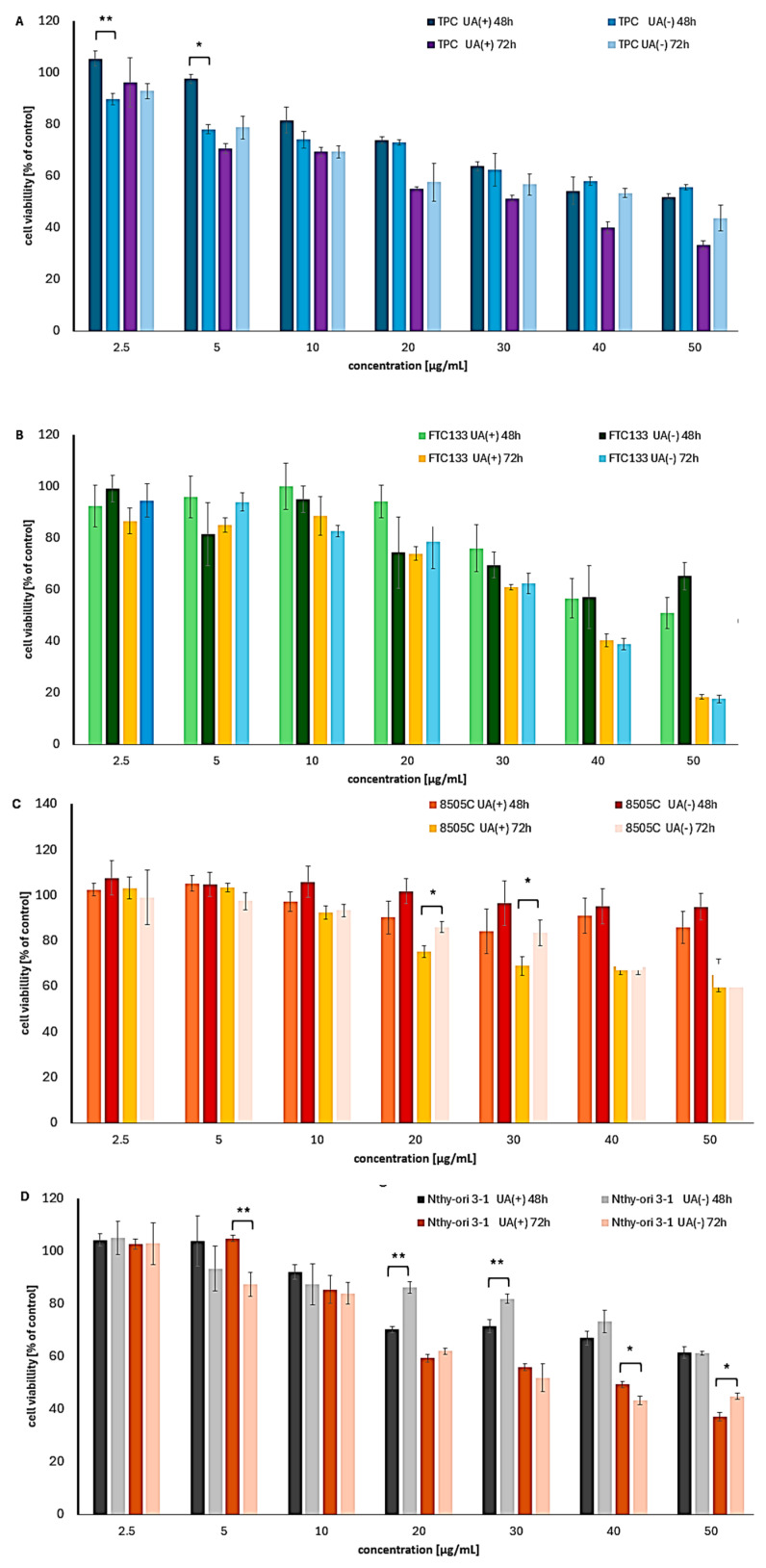
The cytotoxic impact of (+)- and (−)-UA on the viability of (**A**) TPC-1, (**B**) FTC133, (**C**) 8505C thyroid cancer cells, and (**D**) Nthy ori 3-1 normal thyroid cells after 48 and 72 h of exposure. Statistical analysis was performed by one-way analysis of variance (ANOVA), followed by Tukey’s post hoc test, with *p* ≤ 0.05 indicating statistically significant differences. Statistical differences between the enantiomers within the same concentration and exposure time are marked with black clamps, with *p* < 0.05 (*), and *p* < 0.01 (**).

The cytotoxic effects of UA enantiomers on thyroid cancer cells became evident only after 48 and 72 h of treatment, as cell viability remained above 80% following 24 h of exposure (Table S1). Among the tested thyroid cancer cell lines, the most notable results were observed in papillary TPC-1 and follicular FTC133 cells, with IC_50_ values of approximately 30 µg/mL for both enantiomers after 72 h of incubation (Table 3, Figure 4).

However, distinct trends were observed between the two cell lines in terms of time- and dose-dependent responses. In TPC-1 cells, a more pronounced reduction in cell viability was noted after 48 and 72 h of exposure, particularly at concentrations above 40 µg/mL. In contrast, FTC133 cells exhibited a more consistent time-dependent effect. Notably, FTC133 cells were more sensitive to UA enantiomers than TPC-1 cells, especially at lower concentrations.

Anaplastic 8505C cancer cells were the most resistant to both compounds; however, their viability was affected after prolonged exposure and at concentrations exceeding 30 µg/mL. Given that anaplastic thyroid cancer cells are known for their resistance to conventional therapies [27], these findings are noteworthy and warrant further investigation.

Interestingly, no clear predominance of either enantiomer was observed across the thyroid cancer cell lines. To date, no data have been published on the cytotoxic effects of UA enantiomers on thyroid cancer cells, suggesting that our results may indicate a new direction for future research.

### 2.4. Cytotoxic Effect of UA Enantiomers on Breast Cancer Cells

The two breast cancer cell lines examined exhibited distinct responses to UA enantiomers. After 24 h of exposure, the viability of MCF7 cells remained comparable to that of untreated control cells. In contrast, a slightly greater cytotoxic effect was observed in MDA-MB-231 cells, although overall cell viability remained above 65% (Table S1).

**Figure 5:**
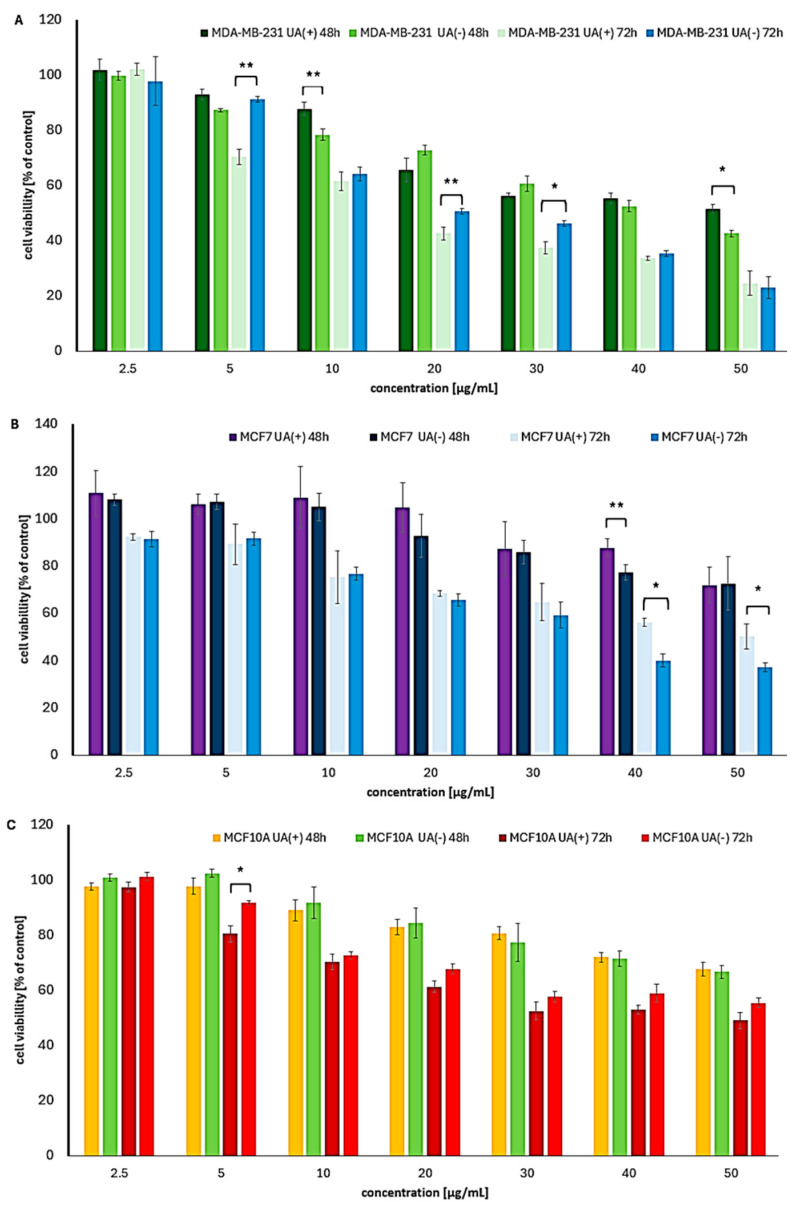
The cytotoxic impact of (+)- and (−)-UA on the viability of (**A**) MDA-MB-231, (**B**) MCF7 breast cancer cells, and (**C**) MCF10A normal breast cells after 48 and 72 h of exposure. Statistical analysis was performed by one-way analysis of variance (ANOVA), followed by Tukey’s post hoc test, with *p* ≤ 0.05 indicating statistically significant differences. Statistical differences between the enantiomers within the same concentration and exposure time are marked with black clamps, with *p* < 0.05 (*), and *p* < 0.01 (**).

Prolonged exposure revealed a higher sensitivity of the triple-negative MDA-MB-231 cells, with IC_50_ values of 15.8 µg/mL and 20.2 µg/mL for (+)-UA and (−)-UA, respectively, after 72 h. In comparison, MCF7 cells, which express the estrogen receptor, were less susceptible to both enantiomers (Table 4, Figure 5).

No clear predominance of either enantiomer was observed in the breast cancer cell lines. To date, only two studies have reported the effects of both UA enantiomers on MCF7 cells. One study indicated comparable activity between the enantiomers [24], while another reported a two-fold greater effect of (−)-UA compared to (+)-UA, with IC_50_ values of 105.4 µM vs. 51.7 µM (equivalent to 36.3 vs. 17.8 µg/mL) after 72 h—findings that align with our results. As for MDA-MB-231 cells, only a few studies have examined the effects of (+)-UA. Reported IC_50_ values include 13.1 µM (4.5 µg/mL) after 72 h [28] and 38.41 µM (13.23 µg/mL) after 24 h [29], both of which indicate stronger cytotoxicity than observed in our study. However, it is important to note that our research is the first to report the cytotoxic potential of (−)-UA in MDA-MB-231 cells. Moreover, the promising results obtained for the triple-negative, clinically aggressive MDA-MB-231 cell line following treatment with usnic acid enantiomers highlight the translational potential of these compounds and may serve as a valuable foundation for future in-depth investigations.

### 2.5. Cytotoxic Effect of UA Enantiomers on Brain Cancer Cells

**Figure 6:**
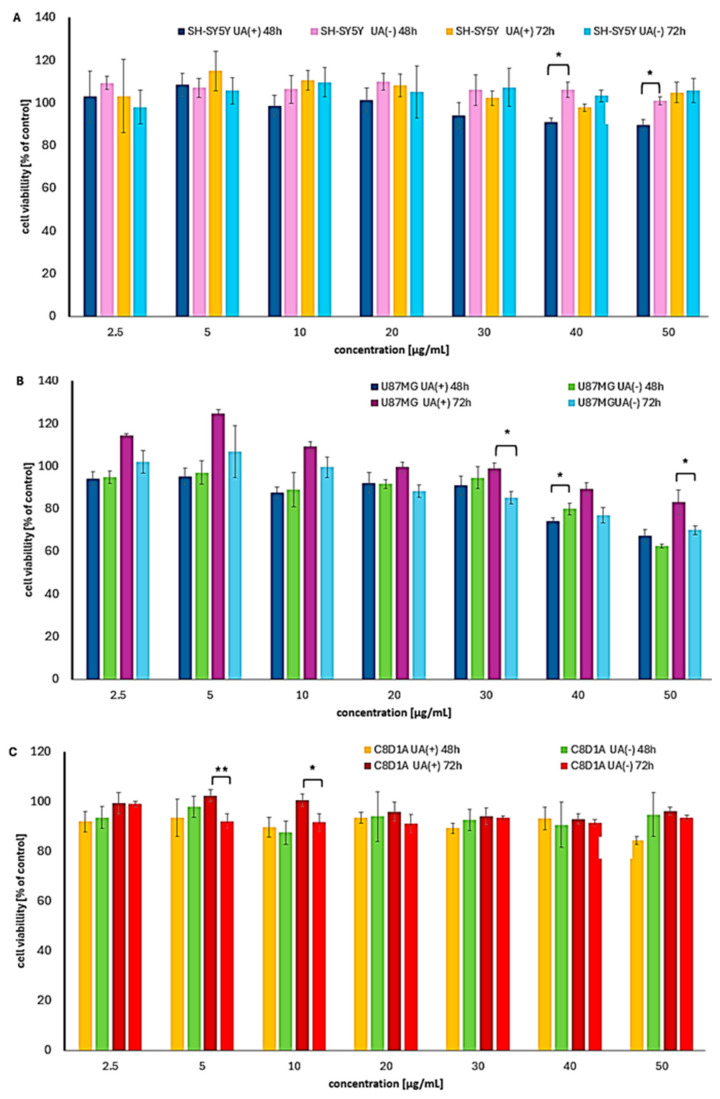
The cytotoxic impact of (+)- and (−)-UA on the viability of (**A**) neuroblastoma SH-SY5Y, (**B**) glioblastoma U87MG cells, and (**C**) C8D1A normal astrocytes after 48 and 72 h of exposure. Statistical analysis was performed by one-way analysis of variance (ANOVA), followed by Tukey’s post hoc test, with *p* ≤ 0.05 indicating statistically significant differences. Statistical differences between the enantiomers within the same concentration and exposure time are marked with black clamps, with *p* < 0.05 (*), and *p* < 0.01 (**).

Among all the cancer cell lines tested, brain cancer cells were the least responsive to UA enantiomers (Table S1, Figure 6). The viability of neuroblastoma SH-SY5Y cells remained unchanged compared to untreated controls, regardless of exposure time. In glioblastoma U87MG cells, only a weak cytotoxic effect was observed, primarily at the highest concentrations tested. Interestingly, the effect was more pronounced after 48 h than after 72 h of exposure. A slight predominance of (−)-UA was noted, though the difference was minimal.

Due to its lipophilic nature, UA is capable of crossing the blood–brain barrier, and although current data are limited, the observed effects are promising [30]. To date, only one study has directly compared the effects of both UA enantiomers on glioblastoma U251 cells, reporting nearly identical IC_50_ values of 19.5 and 19.7 µM (6.7 and 6.8 µg/mL) for (+)-UA and (−)-UA, respectively, after 72 h [23].

Our findings contrast with those of [31], who reported a significantly higher cytotoxic effect of (+)-UA on U87MG cells, with a CC_50_ of 47 µM (16 µg/mL) after 24 h. Conversely, [32] reports an IC_50_ of 41.55 mg/mL after 48 h for the same cell line and enantiomer, suggesting no meaningful activity. These discrepancies highlight the need for further investigation into the effects of UA enantiomers on brain cancer cells.

Interestingly, a single study has reported the cytotoxic effects of (−)-UA on A172 and T98G human glioblastoma cell lines, with IC_50_ values of 31.5 µg/mL and 13 µg/mL, respectively [33]. Data on the effects of (+)-UA on SH-SY5Y neuroblastoma cells are limited. Reported results include 57% cell viability at a concentration of 10 µg/mL [34] and 42% viability at 2 µg/mL [35]. However, no data are currently available regarding the effects of (−)-UA on SH-SY5Y cells.

### 2.6. Cytotoxic Effect of UA Enantiomers on Melanoma Cells

In our previously published study, the effects of UA enantiomers on three human melanoma cell lines were evaluated after 24 and 48 h of incubation [12]. A varied, dose- and time-dependent cytotoxic response was observed, with a clear predominance of (+)-UA, showing nearly a two-fold difference in IC_50_ values compared to (−)-UA.

Among the tested cell lines, the malignant melanoma A375 cells were the most sensitive to (+)-UA, with an IC_50_ of 11.84 µg/mL after 48 h. In contrast, the highly metastatic HTB140 cells exhibited a less pronounced response (IC_50_ of 14.72 µg/mL). Notably, both enantiomers affected the viability of primary melanoma WM793 cells—despite their resistance to doxorubicin (IC_50_ > 100 µg/mL)—with IC_50_ values of 30.05 µg/mL for (+)-UA and 52.09 µg/mL for (−)-UA after 48 h, although the overall effect was moderate.

To date, no other studies have directly compared the cytotoxicity of both UA enantiomers in melanoma cells. However, the same authors have reported the high activity of UA against other melanoma-derived cell lines, including FemX (IC_50_ = 12.72 µg/mL, 72 h), derived from a lymph node metastasis, and 518A2 (IC_50_ = 5.4 µM ≈ 2 µg/mL, 72 h), derived from an unspecified metastatic site [36,37]. Unfortunately, these studies did not specify which enantiomer was used.

### 2.7. Evaluation of Toxicity and Selectivity of UA Enantiomers to Non-Cancerous Cells

To assess the toxicity of UA enantiomers, non-cancerous cell lines corresponding to each cancer panel were included in the study. Where applicable, selectivity indexes (SIs) were calculated to highlight the specific activity of UA enantiomers toward cancer cells. Additionally, data from our previous studies on hepatotoxicity in HepG2 cells [38] and toxicity to non-cancerous skin cell lines [19] were incorporated to provide a broader perspective.

UA enantiomers did not affect normal colon epithelial cells, even after 72 h of exposure, with cell viability remaining above 80% (Table 1, Figure 2D). Although SI could not be calculated in this case, the results suggest the high safety and selectivity of UA enantiomers, especially when compared to their significant cytotoxicity against colon adenocarcinoma cells and the strong toxicity of doxorubicin (IC_50_ = 2.25 µg/mL, 24 h). These findings support further investigation, including additional colon cancer cell lines and potential in vivo studies.

Conversely, UA is known for its hepatotoxic effects. In our recent study, we demonstrated the higher hepatotoxicity of (−)-UA compared to (+)-UA (IC_50_ = 28.2 vs. 16.0 µg/mL and 24.4 vs. 18.3 µg/mL after 48 and 72 h, respectively), although both were significantly less toxic than doxorubicin (IC_50_ = 1.03 µg/mL, 24 h). Importantly, we showed that this toxicity could be mitigated by co-treatment with hepatoprotective agents such as silybin, squalene, and N-acetylcysteine [38]. Based on these findings, we recommend the use of (+)-UA in future studies on gastrointestinal cancers.

In normal prostate epithelial cells, UA enantiomers exhibited notable toxicity (Table 2, Figure 3D), though still lower than that of doxorubicin (IC_50_ = 1.4 µg/mL). A slight predominance of (+)-UA was observed after 72 h. The cytotoxic effect was comparable (48 h) or even greater (72 h) than that observed in PC3 cancer cells, resulting in SI values below 1. However, better selectivity was achieved in DU145 cells, particularly for (+)-UA after 48 h, with SI exceeding 3. This indicates promising selectivity and supports further investigation.

In normal thyroid epithelial cells, UA enantiomers showed considerable toxicity (Figure 4D), with IC_50_ values comparable to those observed in FTC133 and TPC-1 thyroid cancer cells (Table 3), and similar to doxorubicin (IC_50_ = 27.2 µg/mL, 24 h). These findings were further supported by low SI values, mostly below 1.

Normal breast epithelial cells were moderately affected by UA enantiomers, with the most pronounced toxicity observed after 72 h (Table 4, Figure 5C). A slight, though not statistically significant, predominance of (+)-UA was noted. Notably, the SI for (+)-UA in MCF7 cells after 72 h was nearly 3, indicating high selectivity and justifying further research.

For the brain cancer panel, murine astrocytes were used to assess safety. Neither enantiomer affected cell viability, regardless of dose or exposure time (Figure 6C), suggesting high safety compared to doxorubicin (IC_50_ = 2.25 µg/mL, 24 h). However, given the relatively weak cytotoxic effects of UA enantiomers on glioblastoma and neuroblastoma cells, future studies should explore higher doses or combination therapies with cytostatic agents.

In our previous study, we also examined the toxicity of UA enantiomers on human non-cancerous skin cells, including keratinocytes, fibroblasts, and melanocytes. UA enantiomers exhibited relatively low toxicity compared to doxorubicin (IC_50_ = 4.7 µg/mL, 24 h), with effects observed primarily after prolonged exposure. (−)-UA showed higher toxicity, particularly in HaCaT keratinocytes (IC_50_ = 80.82 and 70.12 µg/mL after 48 and 72 h, respectively), while other skin cell lines were more resistant [19].

## 3. Materials and Methods

### 3.1. Chemicals and Cell Culture Media

Both (+)- and (−)-UA were obtained by isolation from *Cladonia arbuscula* and *C. uncialis*, respectively, in our previous studies [39,40], and the details are presented in the Supplementary Material. DMSO, thiazolyl blue tetrazolium bromide (MTT), and doxorubicin were purchased from Merck (Darmstadt, Germany). Cell culture media and supplements: DMEM/F12, DMEM Low Glucose, DMEM High Glucose, MEM, RPMI1640, epidermal growth factor (EGF), insulin, hydrocortisone, cholera toxin, fetal bovine serum (FBS), donor horse serum, trypsin-EDTA solution, penicillin–streptomycin solution, phosphate buffered saline (PBS) were purchased from Merck (Darmstadt, Germany). A multi-detection microplate reader (Multi-Detection Microplate Reader SynergyTM HT—BioTek Instruments Inc., Winooski, VT, USA) was used in the study.

### 3.2. Cell Lines

Cell lines of different origins were used in the study and grouped as follows: prostate panel (human prostate carcinomas: DU145, derived from metastatic site: brain; grade IV PC3, derived from metastatic site: bone; LNCaP, derived from the metastatic site: lymph node; prostate epithelial, PNT2), colon panel (colorectal adenocarcinomas: HT29, originating from a primary tumor; Duke’s type C DLD-1, with the potential to lymph node metastasis; highly metastatic HCT116; colon epithelial cells CCD 841 CoN), thyroid panel (follicular thyroid carcinoma FTC133, anaplastic thyroid carcinoma 8505C, papillary thyroid carcinoma TPC-1, normal thyroid follicular epithelial Nthy-ori 3–1), breast panel (ER-positive breast adenocarcinoma MCF7, ER-negative breast adenocarcinoma MDA-MB-231, breast epithelial MCF10A), brain panel (neuroblastoma SH-SY5Y, glioblastoma U-87 MG, murine astrocytes C8D1A). All cell lines were purchased from Merck (Darmstadt, Germany).

### 3.3. Cell Culture and Treatment

Cells were grown under standard conditions (37 °C, 5% CO_2_) and culture media (DMEM/F12 for PNT2, PC3, FTC133, 8505 C, MDA-MB-231, TPC-1; DMEM Low Glucose for DU145; DMEM High Glucose for HCT116, DLD-1, SH-SY5Y, U87MG, C8D1A; MEM with NEAA for MCF7; MEM for CCD 841 CoN; McCoy’s for HT29; RPMI1640 with sodium pyruvate for LNCaP; DMEM/F12 with 20 ng/mL epidermal growth factor (EGF), 10 μg/mL insulin, 0.5 μg/mL hydrocortisone, 100 ng/mL cholera toxin) for MCF10A), supplemented with 10% fetal bovine serum (FBS) or 5% donor horse serum for MCF10A, and 1% antibiotics solution (10,000 U penicillin and 10 mg streptomycin/mL). The tested substances were prepared as 10 mg/mL stock solutions in DMSO and further diluted in the culture media to the working concentrations. Cells were treated with UA enantiomers at the concentration range of 0–50 µg/mL. The influence of DMSO on cell viability was also examined, with the final concentration not exceeding 0.1%, and no toxic effect was noted.

### 3.4. Viability Assay

The cells were seeded onto 96-well plates at a density of 1.5 × 10^4^ cells/well. After 24 h UA enantiomers were added. Cell viability was determined after 24, 48, and 72 h by the MTT assay, as described previously [41]. Doxorubicin was used as a reference cytostatic drug. All analyses were performed in triplicate; cell viability was expressed as % of control, untreated cells (mean ± SD), and IC_50_ values (concentration at which cell viability is inhibited by 50%), if possible.

### 3.5. Selectivity Index

The selectivity index (SI) was calculated, if possible, by dividing the IC_50_ value obtained for non-cancerous cells line by the IC_50_ values obtained for appropriate cancer cell line, as described previously [42]. In general, the higher the SI value, the more effective and safer a substance is considered. The values above 3 indicate good selectivity.

### 3.6. Statistical Analysis

The data obtained were subjected to statistical analysis using one-way analysis of variance (ANOVA), followed by Tukey’s post hoc test to assess differences between groups. Statistical calculations were performed using STATISTICA v. 13.3 (TIBCO Software Inc., Palo Alto, CA, USA). A *p*-value of ≤0.05 was considered indicative of statistically significant differences among the groups.

## 4. Conclusions

This study was designed to explore and compare the potential application of individual, naturally derived UA enantiomers in cancer chemoprevention or as selective agents targeting specific tumor types. The findings contribute to the development of novel anticancer strategies based on natural products. Overall, (+)-UA demonstrated stronger cytotoxic activity than the (−)-enantiomer, particularly in colon, prostate, and breast cancer cell lines. Thyroid cancer cell lines showed moderate differences between enantiomers, with generally weaker responses.

The cytotoxic effects were more pronounced after 72 h of exposure, while significantly lower activity was observed after 24 h. This suggests a time-dependent mechanism of action and supports a minimum incubation period of 48 h in future studies. Several normal (non-cancerous) cell lines exhibited lower sensitivity to UA, indicating a favorable selectivity profile, especially for the (+)-enantiomer. However, further studies are necessary to conclusively determine the superiority of one enantiomer over the other.

## Figures and Tables

**Table 1:** IC_50_ (µg/mL) values for UA enantiomers for colon cancer and normal cell lines.

Cell Line	UA(+) 48 h	UA(−) 48 h	UA(+) 72 h	UA(−) 72 h
DLD-1	26.1	44.3	19.6	15.4
HCT116	26.4	26.2	10.5	10.7
HT29	>50	>50	>50	32.7
CCD 841 CoN	>50	>50	>50	>50

UA(+) right-handed UA; UA(−) left-handed UA; 48 h, 72 h times of exposure; for cell line abbreviations, see Material and Methods Section.

**Table 2:** IC_50_ (µg/mL) and selectivity index (SI) values for UA enantiomers for prostate cancer and normal cell lines.

Cell Line	UA(+) 48 h	UA(−) 48 h	UA(+) 72 h	UA(−) 72 h
LNCaP	>50	>50	>50	>50
DU145	11.5 (SI 3.25)	34.8 (SI 1.02)	12.1 (SI 1.68)	18.6 (SI 1.49)
PC3	39.6 (SI 0.94)	37.8 (SI 0.93)	19.1 (SI 1.07)	34.8 (SI 0.79)
PNT2	37.4	35.4	20.4	27.8

UA(+) right-handed UA; UA(−) left-handed UA; 48 h, 72 h times of exposure; for cell line abbreviations, see the Material and Methods Section.

**Table 3:** IC_50_ (µg/mL) and selectivity index (SI) values for UA enantiomers for thyroid cancer and normal cell lines.

Cell Line	UA(+) 48 h	UA(−) 48 h	UA(+) 72 h	UA(−) 72 h
8505C	>50	>50	>50	>50
TPC-1	>50	>50	28.2 (SI 1.18)	39.2 (SI 0.74)
FTC133	>50	>50	35.1 (SI 0.95)	35.3 (SI 0.83)
Nthy ori 3-1	>50	>50	33.2	29.3

UA(+) right-handed UA; UA(−) left-handed UA; 48 h, 72 h times of exposure; for cell line abbreviations, see the Material and Methods Section.

**Table 4:** IC_50_ (µg/mL) and selectivity index (SI) values for UA enantiomers for breast cancer and normal cell lines.

Cell Line	UA(+) 48 h	UA(−) 48 h	UA(+) 72 h	UA(−) 72 h
MDA-MB-231	>50	41.4	15.8 (SI 2.83)	20.2
MCF7	>50	>50	>50	33.4
MCF10A	>50	>50	44.8	>50

UA(+) right-handed UA; UA(−) left-handed UA; 48 h, 72 h times of exposure; for cell line abbreviations, see the Material and Methods Section.

## Data Availability

All data are included in the manuscript. For raw data please contact the corresponding author.

## Supplementary Materials

The following supporting information can be downloaded at: https://www.mdpi.com/article/10.3390/molecules30142964/s1, Isolation procedure and identification of usnic acid enantiomers; Table S1: Impact of (+)- and (−)-UA (UA) on viability of cancer and normal cell lines after 24 h of exposure.
