# Supplementary material for: An Evaluation of the Cytotoxicity and Safety Profile of Usnic Acid for a Broad Panel of Human Cancers and Normal Cells with Respect to Its Enantiospecificity
Source: Molecules. 2025 Jul 14;30(14):2964. doi: 10.3390/molecules30142964 (PMC12298648; doi:10.3390/molecules30142964)
Supplement: Supplementary file 1 [file molecules-30-02964-s001.zip › molecules-3723824-supplementary.pdf]

Isolation procedure and identification of usnic acid enantiomers

Briefly, lichen thalli were extracted consecutively with hexane and chloroform in a Soxhlet apparatus. Crystals of the compound, which had been obtained during extract cooling, were purified by recrystallization. The identity and purity of the compound was assessed by measurement of melting point, optical rotation and by HPLC analyses, by comparing Rt and UV spectrum with those of standard (+)-usnic acid (Sigma- Aldrich). Chloroform solution of isolated usnic acid was analyzed by HPLC under following conditions: Dionex HPLC system (PDA detector, Hypersil Gold C-18 column (250 × 4.6 mm), mobile phase consisting of methanol-water-phosphoric acid 80:20:0.9, flow rate: 1.0 mL/min, detection: 240 nm. The enantiomeric form of isolated usnic acid enantiomers was confirmed using a Jasco P-2000 polarimeter.

Optical rotation $[\alpha]_D^{24}$ - 475° (CHCl3) for isolated (-)UA, and -488 for the reference.

Optical rotation $[\alpha]_D^{24}$ + 478° (CHCl3)for isolated (+)UA, and +489 for the reference.

melting point 202–204 for isolates, and 203–204 °C, for the reference

purity 95.2% (+)UA and 95.6% (-)UA

Table S1. Impact of (+)- and (-)-usnic acid (UA) on viability of cancer and normal cell lines. after 24 h of exposure.

Cell viability is presented as % of control. untreated cells ± SD

Concentration µg/mL	colon cells			
DLD-1	UA(+)24	+/-SD	UA(-)24h	+/-SD
50	68.46	1.72	77.98	2.76
40	68.28	1.49	77.75	3.98
30	71.08	2.50	78.35	3.49
20	82.11	1.25	81.25	1.81
10	75.63	1.89	92.05	3.29
5	89.29	1.88	97.99	2.04
2.5	96.51	2.11	100.75	2.67
HCT116	UA(+)24	+/-SD	UA(-)24h	+/-SD
50	61.49	4.91	73.33	4.09
40	67.02	5.64	71.90	0.62
30	68.16	4.15	72.86	3.94
20	71.99	6.48	84.97	4.14
10	85.60	3.57	96.26	4.44
5	103.97	3.10	108.16	1.24
2.5	100.35	3.59	112.65	4.76
HT29	UA(+)24	+/-SD	UA(-)24h	+/-SD

50	72.56	2.67	73.95	3.51
40	74.11	5.47	72.48	3.23
30	77.54	1.35	74.37	2.42
20	84.70	2.33	79.62	3.59
10	88.42	2.22	84.03	2.31
5	91.51	1.48	90.48	1.19
2.5	103.23	8.21	102.45	3.48
CCD 841 CoN	UA(+)24	+/-SD	UA(-)24h	+/-SD
50	96.52	7.15	93.06	6.74
40	95.35	6.60	96.94	1.27
30	101.26	3.72	96.11	4.88
20	111.77	10.58	99.72	8.35
10	99.72	8.91	96.39	4.88
5	103.61	5.91	98.89	3.76
2.5	104.22	7.20	98.61	3.47
	**prostate cells**			
LNCaP	UA(+)24	+/-SD	UA(-)24h	+/-SD
50	69.71	1.76	89.39	9.92
40	69.13	2.17	79.09	5.24
30	71.45	1.76	81.97	6.70
20	75.51	1.65	85.30	5.86
10	79.71	3.54	88.48	4.30
5	81.45	1.09	98.18	2.41
2.5	87.68	1.33	100.45	5.00
DU145	UA(+)24	+/-SD	UA(-)24h	+/-SD
50	67.13	0.85	65.86	2.39
40	68.15	4.14	64.95	0.92
30	72.41	1.34	68.28	2.13
20	87.54	8.79	77.63	1.97
10	86.92	2.72	82.15	0.49
5	94.38	1.28	89.03	1.43
2.5	102.26	2.39	99.84	4.07
PNT2	UA(+)24	+/-SD	UA(-)24h	+/-SD
50	56.52	1.89	54.38	3.61
40	59.70	3.69	68.75	2.53
30	68.67	3.36	74.58	2.66
20	96.96	3.34	83.13	1.57
10	104.00	1.76	91.32	4.22
5	101.11	5.03	99.24	6.94
2.5	112.52	2.27	103.40	3.34
PC3	UA(+)24	+/-SD	UA(-)24h	+/-SD
50	64.83	1.60	68.91	1.24
40	70.10	3.29	69.15	1.64
30	83.08	2.63	76.52	1.50

20	96.62	5.00	89.10	2.79
10	104.63	1.87	91.59	2.12
5	99.30	2.57	94.68	3.66
2.5	100.80	2.77	102.94	2.84
	**breast cell**			
MDA-MB-231	UA(+)24	+/-SD	UA(-)24h	+/-SD
50	69.24	5.40	70.55	2.10
40	67.93	5.11	68.12	7.82
30	77.62	3.90	74.48	6.96
20	81.99	5.98	85.58	3.01
10	87.62	3.06	88.24	2.20
5	95.55	1.91	92.06	1.55
2.5	100.36	4.87	96.79	1.24
MCF7	UA(+)24	+/-SD	UA(-)24h	+/-SD
50	87.78	2.67	91.15	4.66
40	87.41	6.86	106.28	13.07
30	89.38	9.22	109.10	7.36
20	103.95	1.54	106.15	8.06
10	108.15	9.25	110.03	6.29
5	108.77	4.26	113.59	3.57
2.5	106.54	6.02	124.74	10.22
MCF10A	UA(+)24	+/-SD	UA(-)24h	+/-SD
50	94.02	7.65	99.39	1.09
40	97.35	1.18	103.54	1.43
30	97.25	1.67	100.10	5.04
20	97.06	3.89	98.59	8.64
10	94.31	7.19	101.52	4.21
5	98.53	3.85	100.30	2.73
2.5	95.69	2.83	102.83	2.82
	thyroid cell			
8505C	UA(+)24	+/-SD	UA(-)24h	+/-SD
50	108.63	3.02	94.42	11.04
40	102.26	5.72	103.98	4.72
30	99.47	3.99	102.79	3.30
20	102.26	3.83	100.00	3.74
10	98.80	1.59	100.80	3.31
5	103.05	4.22	107.97	3.43
2.5	102.52	3.02	105.18	4.40
Nthy ori 3-1	UA(+)24	+/-SD	UA(-)24h	+/-SD
50	97.71	3.27	98.58	3.37
40	97.29	2.11	96.10	0.65
30	94.51	1.51	95.25	5.83
20	93.96	2.50	93.76	2.66
10	95.69	1.54	94.52	5.34
5	100.56	7.32	99.36	4.70

2.5	97.57	4.27	97.23	4.32
TPC-1	UA(+)24	+/-SD	UA(-)24h	+/-SD
50	87.50	1.83	92.38	2.17
40	91.23	1.19	92.26	2.49
30	95.96	4.32	91.95	4.89
20	97.02	2.60	93.49	1.86
10	105.31	2.16	96.79	0.77
5	105.96	3.63	104.40	3.96
2.5	101.62	6.09	96.85	5.00
FTC-133	UA(+)24	+/-SD	UA(-)24h	+/-SD
50	78.08	9.31	83.25	5.06
40	75.75	7.37	86.67	5.58
30	74.75	5.51	93.33	3.38
20	93.83	4.48	89.56	2.62
10	89.83	5.00	87.63	6.64
5	89.00	6.77	106.14	3.69
2.5	92.33	4.88	108.51	8.51
	**Brain cells**			
C8D1A	UA(+)24	+/-SD	UA(-)24h	+/-SD
50	106.42	7.38	104.75	5.63
40	86.17	12.08	90.58	1.59
30	88.67	2.27	88.42	1.88
20	91.17	2.50	96.00	1.30
10	96.58	1.84	95.67	3.61
5	110.17	8.43	99.17	2.89
2.5	110.33	1.63	100.83	2.02
SHSY5Y	UA(+)24	+/-SD	UA(-)24h	+/-SD
50	96.54	6.11	99.40	4.23
40	92.95	4.96	98.57	2.50
30	95.77	5.17	92.62	6.44
20	96.15	3.05	96.67	3.12
10	97.95	3.20	98.10	2.58
5	97.56	4.08	99.17	3.95
2.5	99.49	3.57	96.79	7.43
U87MG	UA(+)24	+/-SD	UA(-)24h	+/-SD
50	95.00	4.60	98.56	1.22
40	92.73	7.79	106.02	2.54
30	101.76	1.31	111.54	7.45
20	103.75	0.72	101.59	5.09
10	98.61	4.94	101.69	3.24
5	99.21	10.07	102.19	1.14
2.5	103.89	6.46	102.34	2.31
